# Correction: Green synthesis of ZnO nanoparticles using *Justicia adhatoda* for photocatalytic degradation of malachite green and reduction of 4-nitrophenol

**DOI:** 10.1039/d5ra90020d

**Published:** 2025-03-12

**Authors:** Munisha Mahajan, Sanjeev Kumar, Jyoti Gaur, Sandeep Kaushal, Jasvir Dalal, Gurjinder Singh, Mrinmoy Misra, Dharamvir Singh Ahlawat

**Affiliations:** a Department of Physics, Chandigarh University Gharuan, Mohali 140413 India; b School of Basic and Applied Sciences, RIMT University Mandi Gobindgarh 147301 India; c Regional Institute of Education, NCERT Ajmer Rajasthan 305004 India kaushalsandeep33@gmail.com; d Department of Physics, Rajdhani College, University of Delhi Delhi 110015 India jasvirdalal2012@gmail.com; e Department of Electrical and Electronics and Communication Engineering, DIT University Dehradun 248009 India; f Mechatronics Engineering Department, School of Automobile, Mechanical and Mechatronics, Manipal University Jaipur India mrinmoy.mishra@jaipur.manipal.edu; g Department of Physics, Chaudhary Devi Lal University Sirsa Haryana 125055 India

## Abstract

Correction for ‘Green synthesis of ZnO nanoparticles using *Justicia adhatoda* for photocatalytic degradation of malachite green and reduction of 4-nitrophenol’ by Munisha Mahajan *et al.*, *RSC Adv.*, 2025, **15**, 2958–2980, https://doi.org/10.1039/D4RA08632E.

The authors regret that the one of the affiliations (affiliation *f*) was incorrectly shown in the original manuscript. The corrected list of affiliations is as shown here.

The Royal Society of Chemistry apologises for these errors and any consequent inconvenience to authors and readers.

